# Head and Neck Cancer Cell Death due to Mitochondrial Damage Induced by Reactive Oxygen Species from Nonthermal Plasma-Activated Media: Based on Transcriptomic Analysis

**DOI:** 10.1155/2021/9951712

**Published:** 2021-07-06

**Authors:** Chan Oh, Ho-Ryun Won, Woo Seok Kang, Dae-Woong Kim, Seung-Nam Jung, Mi Ae Im, Lihua Liu, Yan Li Jin, Yudan Piao, Hae Jong Kim, Yea Eun Kang, Min Joung Lee, Jun Young Heo, Sangmi Jun, Nam Suk Sim, Jeong Ho Lee, Kunho Song, Young Il Kim, Jae Won Chang, Bon Seok Koo

**Affiliations:** ^1^Department of Medical Science, Chungnam National University College of Medicine, Daejeon 35015, Republic of Korea; ^2^Department of Otolaryngology-Head and Neck Surgery, Chungnam National University College of Medicine, Daejeon 35015, Republic of Korea; ^3^Department of Plasma Engineering, Korea Institute of Machinery & Materials, Daejeon 34103, Republic of Korea; ^4^Division of Endocrinology and Metabolism, Department of Internal Medicine, Chungnam National University College of Medicine, Daejeon 35015, Republic of Korea; ^5^Department of Biochemistry, Chungnam National University, Daejeon 34134, Republic of Korea; ^6^Center for Research Equipment, Korea Basic Science Institute, Cheongju 34133, Republic of Korea; ^7^Graduate School of Medical Science and Engineering, KAIST, Daejeon 34141, Republic of Korea; ^8^Department of Radiation Oncology, Chungnam National University College of Medicine, Daejeon 35015, Republic of Korea

## Abstract

Mitochondrial targeted therapy is a next-generation therapeutic approach for cancer that is refractory to conventional treatments. Mitochondrial damage caused by the excessive accumulation of reactive oxygen species (ROS) is a principle of mitochondrial targeted therapy. ROS in nonthermal plasma-activated media (NTPAM) are known to mediate anticancer effects in various cancers including head and neck cancer (HNC). However, the signaling mechanism of HNC cell death via NTPAM-induced ROS has not been fully elucidated. This study evaluated the anticancer effects of NTPAM in HNC and investigated the mechanism using transcriptomic analysis. The viability of HNC cells decreased after NTPAM treatment due to enhanced apoptosis. A human fibroblast cell line and three HNC cell lines were profiled by RNA sequencing. In total, 1 610 differentially expressed genes were identified. Pathway analysis showed that activating transcription factor 4 (ATF4) and C/EBP homologous protein (CHOP) were upstream regulators. Mitochondrial damage was induced by NTPAM, which was associated with enhancements of mitochondrial ROS (mtROS) and ATF4/CHOP regulation. These results suggest that NTPAM induces HNC cell death through the upregulation of ATF4/CHOP activity by damaging mitochondria via excessive mtROS accumulation, similar to mitochondrial targeted therapy.

## 1. Introduction

Head and neck cancer (HNC) is a common cancer that occurs in more than 650 000 people annually worldwide [1]. The regional mortality rate of lip and oral cavity cancer, a representative type of HNC, ranges from 3.9 to 8.7% in men and is currently increasing [1–3]. Despite improvements in diagnosis and treatment methods in recent decades, the 5-year survival rate of advanced HNC remains <50% [2]. In addition, HNC has a higher mortality rate than other types of advanced cancer. Therefore, studies concerning new treatment methods or drugs for HNC are actively ongoing in various fields. In this regard, cancer mitochondria had been broadly used as the target therapy due to the discriminating structure and function between cancer cells and normal cells, such as oxidative stress, the membrane potential (*Δψ*m), and metabolic activity [4]. Mitochondrial damage caused by the excessive ROS is a principal type of mitochondrial targeted therapy.

Plasma is an ionized gas that consists of electrons, ions, radicals, and energetic photons that are generated when energy is applied to a gaseous material [5–7]. Plasma medicine, which entails the clinical use of nonthermal atmospheric pressure plasma (NTP), is a rapidly growing area that could significantly provide new therapeutic applications. NTP reportedly has medical effects such as hemostasis, tissue regeneration, and cancer cell death [8–11]. In particular, NTP has anticancer effects on cervical cancer, pancreatic cancer, thyroid cancer, and HNC [6, 12–15]. However, probe-type gaseous NTP is affected by permeability depending on the application site, and there are limitations for application to internal organs [16]. To address current limitations of existing NTP treatment methods, a liquid form NTP has been devised and its effect has been proven through several studies [9, 10, 17]. Recently, liquid form NTP showed effective anticancer potential against tumor located inside the body, because its reactive oxygen species generated in the gas phase easily enter the cell membrane and destroy the cells [18]. However, many mechanisms underlying biological effects of liquid form NTP have not been elucidated.

In recent years, medical genetics research has focused on gene expression mechanisms and resulting processes in various biological samples. Here, we generated a liquid type of nonthermal plasma-activated media (NTPAM) and investigated its effects on HNC cell lines. In addition, we assessed gene expression before and after NTPAM treatment through RNA sequencing, then confirmed changes through in vitro and in vivo experiments. Our findings demonstrate an important mechanism underlying the anticancer effects of NTPAM on HNC.

## 2. Materials and Methods

### 2.1. Cell Lines and Cultures

Primary normal human fibroblasts, donated by Professor J.H. Lee (Chungnam National University, Daejeon, Republic of Korea), were used as normal epithelial cells. Human fibroblasts were cultured in Dulbecco's modified Eagle's medium (DMEM; WELGENE Inc., Republic of Korea) with 10% fetal bovine serum and 100 U/mL penicillin-streptomycin (Gibco, Gaithersburg, MD, USA). SNU1041 (squamous cell carcinoma of the hypopharynx) and SNU1076 (squamous cell carcinoma of the larynx) cell lines were obtained from the Korean Cell Line Bank (Seoul, Republic of Korea). SNU1041 and SNU1076 were maintained according to KCLB recommendations. The SCC25 (squamous cell carcinoma of the oral tongue) cell line was obtained from the American Type Culture Collection (Manassas, VA, USA). SCC25 was maintained in DMEM : Nutrient Mixture F-12 (WELGENE Inc.) with supplements identical to those of the other cell lines.

### 2.2. Generation of NTPAM

Each medium was treated using an NTP jet generated by a device composed of a quartz tube (outer diameter, 6 mm; inner diameter, 4 mm) with two electrodes (an inner stainless-steel tube electrode and an outer ground ring electrode). The inner tube electrode was also placed as a gas inlet. A high-voltage amplifier supplied input power (a few kV at 20 kHz) to the device. Discharge power varied from 10 to 24 W. The plasma jet was positioned perpendicularly over the medium in a flask, and the gas flowed from the inside of the device toward the medium surface. Helium (He) (4 standard liters per minute (slpm)) and oxygen (O_2_) (1 standard cubic centimeters per minute (sccm)) were used as carrier gases. For NTPAM preparation, NTP was exposed to the culture media (DMEM, RPMI 1640, and DMEM : Nutrient Mixture F-12) for various activation times, thus controlling the concentration of NTPAM. The distance between the plasma device and the bottom of the culture media was maintained at approximately 1 cm. NTPAM was prepared with 60 mL of culture media [19]. The specifications of NTPAM according to the NTP generation time are shown in Figure S1.

### 2.3. Cell Viability Assay

In vitro cell viabilities were estimated by the water-soluble tetrazolium salt-1 (WST-1) assay (Roche Diagnostics, Indianapolis, IN, USA). Cells were seeded in 96-well plates at 8 000 cells/well in 100 *μ*L of the complete media. After cells had been incubated for 24 h, media were replaced with NTPAM. The treated cells were incubated at 37°C for 24 h with 5% CO_2_. WST-1 reagents were then added (10 *μ*L/well) and incubated for 1 h. Plates were thoroughly shaken for 1 min, and the absorbance of formazan product at 450 nm was recorded by a microplate reader.

### 2.4. Annexin V-Fluorescein Isothiocyanate (FITC)/Propidium Iodine (PI) Assay

Annexin V-FITC and PI staining was performed, and cells were detected using a flow cytometer (LSRFortessa X-20; BD Biosciences, Brea, CA, USA) with an Alexa Fluor™ 488 Annexin V/Dead Cell Apoptosis Kit (Invitrogen, Carlsbad, CA, USA), in accordance with the manufacturer's recommendation. FlowJo software version 10 (Tree Star, Ashland, OR, USA) was used to assess the percentages of cells in four populations.

### 2.5. RNA Preparation for Sequencing

In total, six samples per cell line were divided into three control samples and three NTPAM treatment samples. Total RNA extraction and construction of libraries were performed as described previously [19]. RNA quality involves both purity and integrity. We checked RNA purity 260/280 ratio. Ideally, the 260/280 ratio for RNA should be approximately 2.0. The RNA integrity number (RIN) is a parameter for assigning integrity values to RNA computation. We confirm total RNA integrity using an Agilent Technologies 2100 Bioanalyzer with an RIN value greater than or equal to 7.

### 2.6. Bioinformatics Transcriptome Analysis

Quality control analysis of raw reads obtained through sequencing was performed using FastQC (version 0.11.5; http://www.bioinformatics.babraham.ac.uk/projects/fastqc/). Preprocessing of raw data was performed using Trimmomatic software (0.32; http://www.usadellab.org/cms/?page=trimmomatic), and trimmed reads were mapped to a reference genome (UCSC Homo sapiens reference genome; GRCh37/hg19) using HISAT2 (version 2.0.5; https://daehwankimlab.github.io/hisat2/). After read mapping, transcript assembly was performed using StringTie software (version 1.3.3b; https://ccb.jhu.edu/software/stringtie/). Expression profile values were then obtained for each sample for known transcripts, and read counts and fragments per kilobase per million mapped reads (FPKM) values were determined. For differentially expressed gene analysis, genes with fold change (FC) ≥ 2 or ≤ -1.6 and independent *t*-test raw *p* values < 0.05 were extracted using FPKM analysis. Heat maps were generated with PermutMatrix (version 1.9.3; http://www.atgc-montpellier.fr/permutmatrix/).

Gene ontology analysis was performed by using gene ontology software (http://geneontology.org/). To include only significant results, the false discovery rate threshold was set to *p* < 0.05. Pathway analysis was performed using Ingenuity Pathway Analysis (IPA) (Qiagen). Genes related to canonical pathways extracted through the Ingenuity Pathway Knowledge Base were further analyzed.

### 2.7. RNA Isolation and Real-Time qPCR

Total RNA was extracted using a RNeasy Mini Kit50 (Qiagen). cDNA was synthesized with 1 *μ*g total RNA and TOPscriptTMRTDryMIX (Enzynomics, Daejeon, Korea), in agreement with the manufacturer's instructions. Amplification was carried out using SFCgreen™-Cyan qPCR Master Mix (2X)-Low ROX (SFC probe, Giheung-gu, Republic of Korea). Melting curve analysis was performed. PCR primer sequences are shown in supplementary Materials Table S1.

### 2.8. siRNA Transfection

Cells were seeded in six-well plates at 3 × 10^5^ cells/well. After 24 h of incubation, cells were transfected with 60 nmol siRNA using Lipofectamine RNAi MAX reagent (Invitrogen). Negative siRNA was used as a negative control. ATF-4 siRNA (sc-35112, Santa Cruz Biotechnology, Santa Cruz, CA, USA) was a pool of three unique siRNA duplexes with the following sequences shown in supplementary Materials Table S2.

### 2.9. Western Blot

Cells were lysed using RIPA buffer with a protease inhibitor cocktail (Roche Applied Science, Vienna, Austria). Total protein was quantified using a Pierce BCA Protein Assay Kit (Thermo Fisher Scientific, Waltham, MA, USA). Samples were separated by SDS-PAGE and transferred to PVDF membranes (Millipore, Burlington, MA, USA). Membranes were blocked in TBS with Tween containing 5% skim milk. They were then incubated with specific primary antibodies on a gentle shaker overnight at 4°C. The following primary antibodies were used for Western blotting: antibodies against poly (ADP-ribose) polymerase (PARP), Caspase 3, cytochrome c oxidase (Cox) IV, Succinate dehydrogenase iron-sulfur subunit B (SDHB), ATF4, CHOP, and *β*-actin (all 1 : 1000; Cell Signaling Technology, Danvers, MA, USA); Adenosine triphosphate (ATP) 5A and ubiquinol-cytochrome c reductase core protein 2 (UQCRC2) (both 1 : 1000; Abcam, Cambridge, UK); NADH-ubiquinone oxidoreductase chain 6 (ND6) (1 : 1000; Biocompare, San Francisco, CA, USA); and B-cell lymphoma 2 (Bcl-2) (1 : 1000; Santa Cruz Biotechnology). Membranes were incubated with secondary antibodies on a gentle shaker for 1 h at room temperature. Signals were observed using an ECL kit (Bio-Rad Laboratories, Hercules, CA, USA). Images were quantified with ImageJ software (version 1.53a; National Institutes of Health, Bethesda, MD, USA), and the normalization results were presented as numbers in the image.

### 2.10. Oxygen Consumption Rate (OCR) Measurement

The OCR was determined using a Seahorse XF-24 analyzer (Seahorse Bioscience Inc., North Billerica, MA, USA). Briefly, thyroid cells were grown in XF-24 plates (2 × 10^4^ cells in 200 *μ*L of growth medium per well) and incubated in an incubator at 37°C with 5% CO_2_ for 24 h. A sensor cartridge was positioned into calibration buffer supplied by Seahorse Bioscience and incubated at 37°C in an incubator without CO_2_ for 24 h before the experiment. Immediately before measurement, the cells were washed and incubated in assay medium at 37°C for 1 h in an incubator without CO_2_. Oligomycin A (20 *μ*g/mL), an ATP synthase (complex V) inhibitor, was injected to measure cellular ATP production. Carbonyl cyanide m-chlorophenyl hydrazine (optimized concentration, 50 *μ*M), an uncoupling agent, was used to measure maximal respiration by disrupting the mitochondrial membrane potential. Rotenone (20 *μ*M), a complex I inhibitor, was injected to restrain mitochondrial respiration and allow the calculation of nonmitochondrial respiration. The OCR was shown by a sensor cartridge and calculated using Seahorse XF-24 software.

### 2.11. Transmission Electron Microscopy

Cells were pelleted via centrifugation and fixed in 2.5% glutaraldehyde in 0.1 M phosphate buffer (pH 7.4) at 4°C. After 2 h, the pellets were rinsed three times in 0.1 M phosphate buffer and then postfixed in 1% osmium tetroxide at 4°C for 1–2 h, followed by three washes in phosphate buffer. The pellets were dehydrated through a series of ethanol solutions and exposed to two changes of propylene oxide. After the dehydration process, the pellets were infiltrated with 2 : 1, 1 : 1, and 1 : 2 mixtures of EMbed 812 resin and propylene oxide for 1 h each time. They were finally embedded in 100% EMbed 812 resin. After the resin had been polymerized for 24–48 h at 60°C, ultrathin plastic sections (80 nm thick) were cut at room temperature using a Leica EM UC6 ultramicrotome (Leica Microsystems GmbH, Wetzlar, Germany) and collected on 200-mesh carbon-coated grids. The grids were poststained with 2% uranyl acetate and 1% lead citrate at room temperature for 15 and 5 min, respectively. A Zeiss LEO912AB 120 kV transmission electron microscope (Carl Zeiss, Oberkochen, Germany) and a FEI Tecnai G2 Spirit Twin 120 kV transmission electron microscope (FEI Company, Columbia, MD, USA) were used for transmission electron microscopy observations.

### 2.12. Mitochondrial ROS (mtROS) Measurement

The MitoSOX fluorescent probe (Thermo Fisher Scientific) was used to assess mitochondrial superoxide. Cells were cotreated with or without NTPAM and N-acetyl cysteine (NAC) (2 and 5 mM, respectively), then incubated at 37°C for 24 h. After treatment, cells were stained with MitoSOX (5 *μ*M) in Hank's balanced salt solution for 15 min. Fluorescence-stained cells (2 × 10^5^) were analyzed via flow cytometry (LSRFortessa X-20; BD Biosciences), and FlowJo software version 10 (Tree Star) was used to analyze the proportions of cells.

### 2.13. Xenograft Animal Model and Experimental Protocol

Ten healthy male BALB/c nude mice (Saeronbio, Uiwang-si, Republic of Korea) were used in this experiment. The experimental procedures were allowed by the Chungnam University School of Medicine Animal Experiment Ethics Committee (202006A-CNU-112). Ten mice were randomly separated into a control group and NTPAM treatment group. SNU1041 cells (5 × 10^6^ cells/mL) were prepared and injected subcutaneously into the upper thigh of the hind legs of each mouse. Gross tumor formation was confirmed on the 10th day after injection. Next, 100 *μ*L intratumoral injections of medium in the control group and NTPAM in the experimental group were administered once daily for 11 days. The same amounts of food and water were provided to each mouse, and gross tumor volume was measured at 3-day intervals after treatment. After 3 weeks of tumor cell injection, mice were sacrificed and the tumors were dissected.

### 2.14. Histological and Immunohistochemical Analysis

Excised tumor specimens were fixed in 4% paraformaldehyde solution (Thermo Fisher Scientific). Paraffin blocks were prepared using a standard paraffin sample preparation method; then, tissues were stained with hematoxylin and eosin using a standard protocol. Immunohistochemical analysis was performed as described previously [19]. Sections were incubated with antibodies against Ki-67 (1 : 500; Bethyl Laboratories, Cambridge, UK), ATF4 (1 : 200; Santa Cruz Biotechnology), and CHOP (1 : 200; Cell Signaling Technology). Automatic digital slide scanner (Pannoramic MIDI, 3DHISTECH, Budapest, Hungary) was used to analyze the image. Three fields were randomly selected from each sample, and their optical densities were quantified using ImageJ software (version 1.53a; National Institutes of Health).

### 2.15. Statistics

All experiments were conducted three times, and the mean values were analyzed. Statistical analysis and graphical plots were assessed using Prism 8.0.1 (GraphPad). Data are expressed as means ± standard deviations, and the significance of between-group differences was evaluated using two-tailed Student's *t*-tests. *p* < 0.05 was considered to reveal statistical significance (^∗^*p* < 0.05; ^∗∗^*p* < 0.01; and ^∗∗∗^*p* < 0.001).

## 3. Results

### 3.1. NTPAM Induced Apoptotic Cell Death in HNC Cell Lines

**Figure 1:**
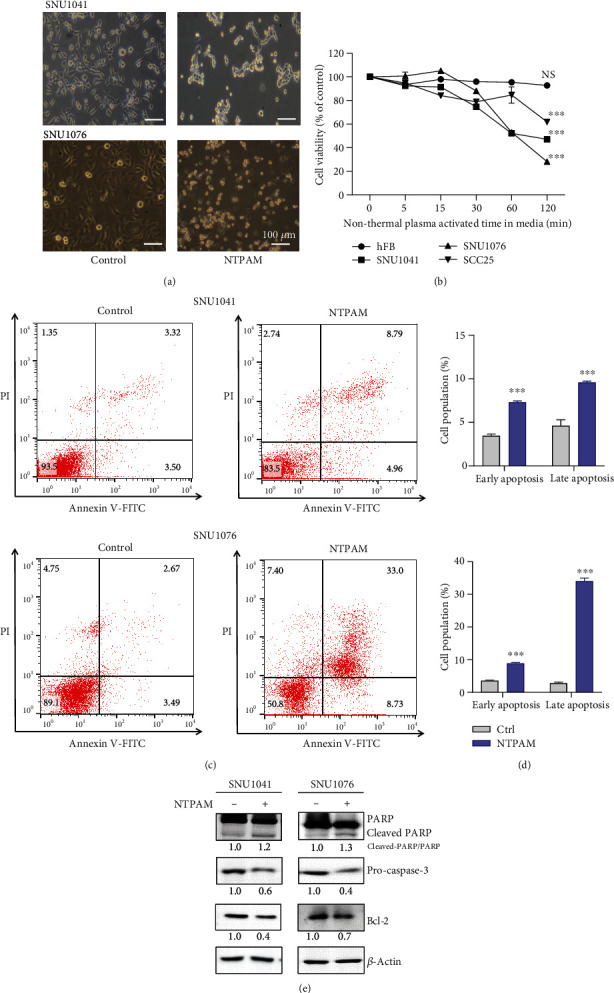
NTPAM induced apoptotic cell death in HNC cell lines. (a) Microscopic evaluation of HNC cell morphology after treatment with NTPAM. (b) Cell viability was analyzed using the WST-1 assay after treatment with NTPAM generated by activation of NTP for 2 h. NTPAM significantly reduced the viabilities of HNC cell lines in an NTP activation-dependent manner. (c, d) Apoptosis was analyzed using the Annexin V-FITC/PI assay. NTPAM significantly enhanced apoptotic cell death in SNU1041 and SNU1076 cell lines. NTPAM was generated by activation of NTP for 2 h and added to cultures for 24 h. (e) NTPAM reduced pro-Caspase 3/Bcl-2 expression and induced cleaved-PARP expression in HNC cell lines, as determined by Western blotting. NS: not significant; ^∗∗∗^*p* ≤ 0.001.

First, we investigated whether NTPAM affected the cell viability of HNC cell lines. Optical microscopy revealed that the number of cells decreased when the SNU1041 and SNU1076 HNC cell lines were treated with NTPAM produced by 2 h of NTP activation (Figure 1(a)). Before and after NTPAM treatment, the WST-1 assay was used to evaluate the viabilities of normal control cells (human fibroblasts) and three HNC cell lines (SNU1041, SNU1076, and SCC25). Cytotoxicity was not observed in the normal control cell line, but cell viability was significantly reduced in HNC cell lines in a manner dependent on NTP activation time (Figure 1(b)). All subsequent experiments were conducted under conditions involving 24 h of incubation with NTPAM that was generated by 2 h of NTP activation. After NTPAM treatment, Annexin V-FITC/PI analysis showed statistically significant enhancements of the fractions of cells corresponding to early apoptotic cells (FITC+/PI-) and late apoptotic cells (FITC+/PI+) (Figures 1(c) and 1(d)).  Figure 1(e) shows apoptosis-related markers assessed after exposure to NTPAM. Notably, the protein levels of cleaved PARP increased, whereas those of pro-Caspase 3 and Bcl-2 decreased. These results suggested that NTPAM selectively induces apoptosis in HNC cells.

### 3.2. Transcriptomic Analysis of HNC Cells after NTPAM Treatment

**Figure 2:**
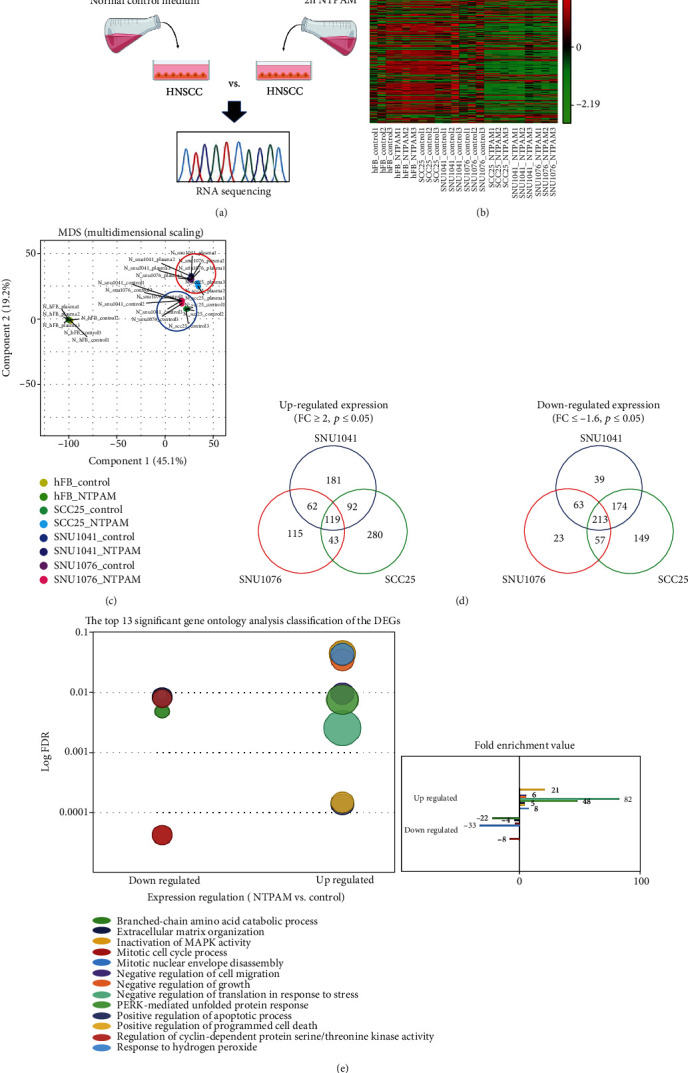
Transcriptomic analysis of HNC cells after NTPAM treatment. (a) Schematic figure of sample preparation process for RNA sequencing. (b) Heat map of one-way hierarchical clustering revealed 1 612 differentially expressed genes (*p* ≤ 0.05). (c) Multidimensional scaling demonstrated the level of similarity of individual datasets. The types of cells are shown by distinct colors. HNC cell lines were similarly clustered and shifted before and after NTPAM treatment, respectively (NTPAM-treated groups of HNC cell lines: red circle, nontreated groups of HNC cell lines: blue circle). (d) Venn diagram demonstrated differentially expressed genes on the basis of RNA sequencing results (FC ≥ 2 or ≤ -1.6, *p* ≤ 0.05). (e) Gene ontology analysis results. The top 13 significantly up- and downregulated pathways in HNC cells after NTPAM treatment.

A normal control cell line (human fibroblasts) and three HNC cell lines (SNU1041, SNU1076, and SCC25) were divided into NTPAM-treated and nontreated groups, and three samples of each group were collected. RNA sequencing was conducted to determine the effects of NTPAM on the transcriptome of each cell line (Figure 2(a)). In total, 1 610 differentially expressed genes (DEGs) were identified depending on NTPAM treatment status (Figure 2(b)). Based on these data, the level of similarity was expressed using multidimensional scaling (Figure 2(c)). In the human fibroblast cell line, there were no noticeable changes in gene expression according to NTPAM treatment status. However, as shown in Figure 2(c), comparisons between the NTPAM-treated (red circle) and nontreated groups (Blue circle) of HNC cell lines showed consistent shifts after NTPAM treatment. Up- and downregulated genes were identified according to NTPAM treatment status in each cell line using FC ≥ 2 or ≤ -1.6 and *p* ≤ 0.05. Synthesis of the results revealed that 119 genes were upregulated and 213 genes were downregulated (Figure 2(d)). Gene ontology analysis was then performed to derive the biological processes and molecular functions of the identified genes, and the top 13 functional processes were identified. The findings showed that NTPAM treatment regulated mechanisms related to cell death and cell cycle. In addition, there was a significant association with the regulation of protein kinase R-like endoplasmic reticulum kinase (PERK), known as an endoplasmic reticulum (ER) stress sensor. This regulation was closely related to an altered cell stress response (Figure 2(e)).

### 3.3. IPA of Gene Expression Profiles in HNC after NTPAM Treatment

**Figure 3:**
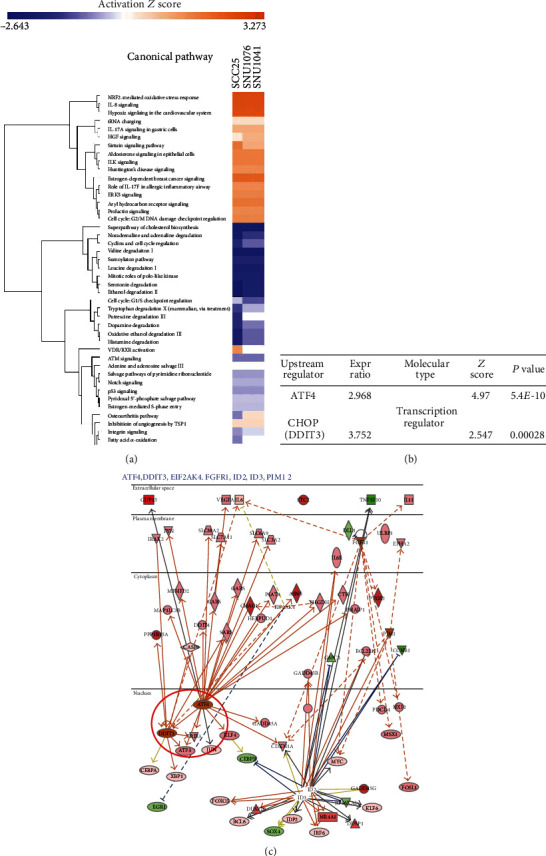
IPA of gene expression profiles in HNC after NTPAM treatment. (a) Top relevant canonical pathways associated with NTPAM treatment by core analysis. (b) List of most potent transcription regulators through upstream regulator analysis. ATF and CHOP (DDIT3) were identified as the most relevant upstream regulators with NTPAM treatment. (c) String network of mechanical signaling pathways related to ATF4 and CHOP.

IPA was performed to identify the mechanism and key factors that may be linked to the DEGs identified in the previous experiment. IPA confirmed the molecular pathways that were up- or downregulated in NTPAM-treated and nontreated groups (Figure 3(a)). Additionally, relevant transcriptional regulators were identified through upstream regulator analysis. In particular, ATF4 and DNA damage-inducible transcript 3 (DDIT3), also known as CHOP, were identified as the most relevant upstream regulators (Figure 3(b)). These results confirmed that ATF4 and CHOP were major upstream transcriptional regulators of the network of mechanical signalling pathways that differed between NTPAM-treated and nontreated groups (Figure 3(c)).

### 3.4. ATF4 And CHOP Are Key Regulators of NTPAM-Induced HNC Cell Death

Transcriptomic analysis indicated that NTPAM induced upregulation of the PERK-related unfolded protein response pathway in HNC cell lines. This suggests that NTPAM may cause cell death due to ER stress. In addition, overexpression of the ATF4/CHOP mechanism (identified through upstream regulator analysis) is reportedly induced by a persistent state of ER stress and promotes apoptotic cell death [20]. Therefore, we presumed that the regulation of ATF4 and CHOP via NTPAM was a key factor in HNC cell death.

**Figure 4:**
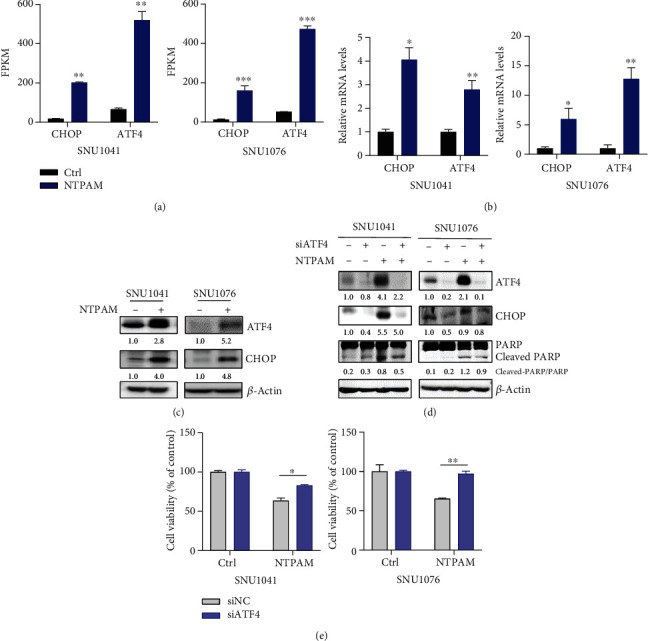
ATF4 and CHOP are key regulators of NTPAM-induced HNC cell death. (a) Analysis of FPKM values of ATF4 and CHOP using RNA sequencing data. Significantly elevated ATF4 and CHOP gene expression levels after NTPAM treatment. (b) Relative expression levels of ATF4 and CHOP mRNA were confirmed by real-time quantitative PCR. NTPAM treatment enhanced the mRNA expression levels of ATF4 and CHOP. (c) Protein expression levels of ATF4 and CHOP in SNU1041 and SNU1076 cell lines were assessed by Western blotting. The protein expression levels of ATF4 and CHOP increased upon NTPAM treatment. (d) After ATF4 knockdown with siRNA, changes in CHOP and PARP protein levels were evaluated. NTPAM treatment increased CHOP and cleaved-PARP levels, but these changes were mitigated by ATF4 knockdown. (e) WST-1 assays showed that the reduced cell viability after NTPAM treatment was alleviated by ATF4 interference. ^∗^*p* ≤ 0.05; ^∗∗^*p* ≤ 0.01; ^∗∗∗^*p* ≤ 0.001.

RNA sequencing demonstrated that the FPKM and relative mRNA expression values of both ATF4 and CHOP significantly increased after NTPAM treatment (Figures 4(a) and 4(b)). As shown in Figure 4(c), the protein levels of ATF4 and CHOP also increased after NTPAM treatment. ATF4 interference with siRNA led to the reduction of NTPAM-induced ATF4 overexpression, as well as the reduction of CHOP expression (Figure 4(d)). The NTPAM-induced expression of cleaved-PARP, an apoptosis-related marker, was also reduced (Figure 4(d)). Cell viability analysis revealed a pattern in which NTPAM-induced apoptosis was significantly diminished after ATF4 knockdown (Figure 4(e)). These results suggested that NTPAM induced HNC cell death through the regulation of ATF4 and CHOP.

### 3.5. NTPAM Induces Mitochondrial Damage in HNC Cells

Mitochondria play an important role in cellular metabolism, thus influencing the survival and death of cancer cells [21]. Recently, ATF4 has been identified as a major regulator of the mitochondrial stress response [22]. Therefore, we speculated that the phenomenon of NTPAM-induced HNC cell death may be related to mitochondrial stress response and damage.

**Figure 5:**
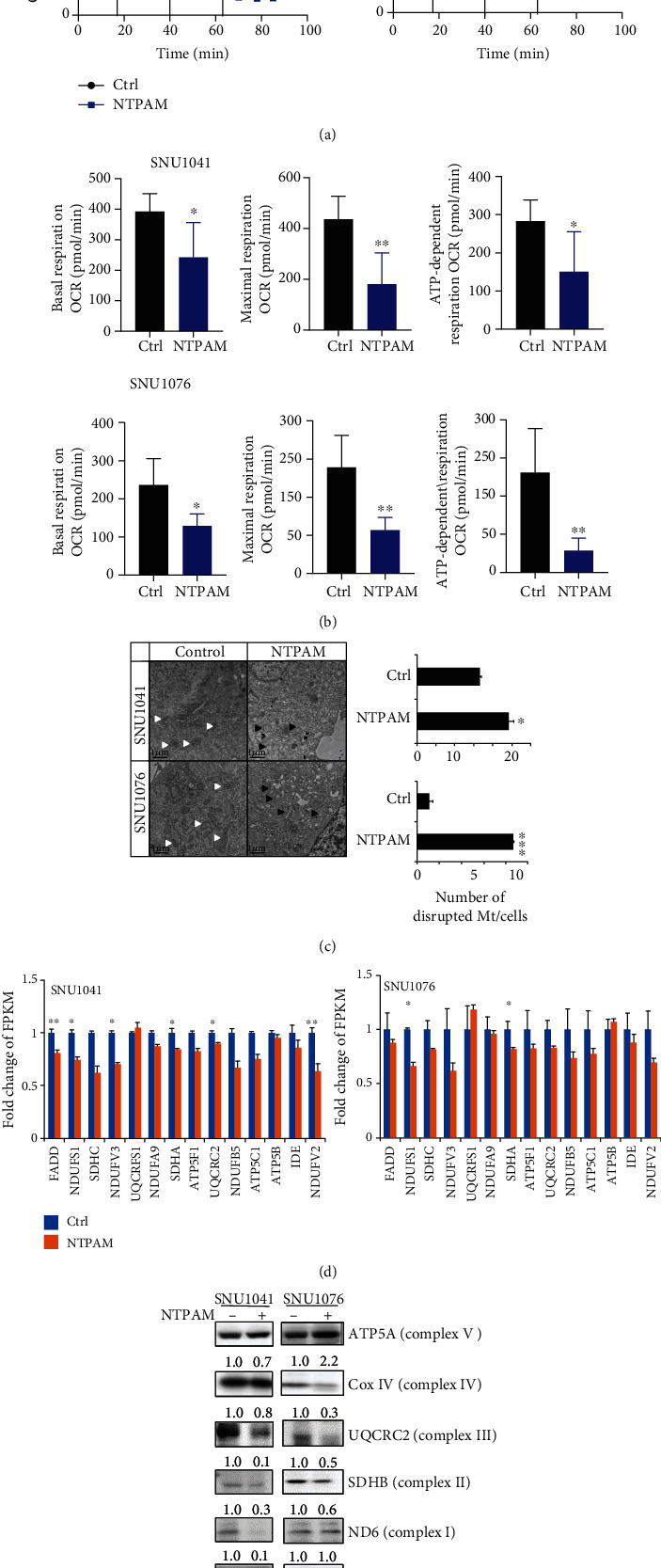
NTPAM induces mitochondrial damage in HNC cells. (a, b) OCR measurement by Seahorse XF-24 analyzer in HNC cell lines (SNU1041 and SNU1076). A significant reduction in OCR was evident after NTPAM treatment. (c) Transmission electron microscopic examination of HNC cell lines before and after NTPAM treatment. Normal mitochondria were disrupted after NTPAM treatment (normal mitochondria: white arrow, dysmorphic mitochondria: black arrow). (d) FPKM values of the oxidative phosphorylation-related genes of SNU1041 and SNU1076 in RNA sequencing data. In SNU1041, the FPKM values of FADD, NDUFS1, NDUFV3, SDHA, UQCRC2, and NDUFV2 were significantly reduced after NTPAM treatment. In SNU1046, the FPKM values of NDUFS1 and SDHA were significantly reduced. (e) Western blotting analyses of oxidative phosphorylation complex protein levels in HNC cell lines. Protein levels of Cox IV (complex IV), UQCRC2 (complex III), and SDHB (complex II) were reduced after NTPAM treatment in SNU1041 and SNU1076 cell lines. ^∗^*p* ≤ 0.05; ^∗∗^*p* ≤ 0.01; ^∗∗∗^*p* ≤ 0.001.

To assess mitochondrial function in HNC cell lines after NTPAM treatment, we analyzed the OCR. Compared with nontreated cells, the treated cells exhibited reduced mitochondrial function (Figures 5(a) and 5(b)) and elevated numbers of disrupted mitochondria (Figure 5(c)). We also examined the human fibroblasts when treated with NTPAM. OCR before and after NTPAM treatment did not differ in human fibroblasts (Figures S2a and b). Furthermore, RNA sequencing analysis showed that the FPKM values of mitochondrial oxidative phosphorylation-related genes tended to decrease after NTPAM treatment. In particular, NDUFS1 and SDHA were significantly reduced in both SNU1041 and SNU1076 cell lines (Figure 5(d)). In addition, the protein levels of enzymes involved in oxidative phosphorylation were examined by Western blotting. After NTPAM treatment, the levels of the Cox IV (complex IV), UQCRC2 (complex III), and SDHB (complex II) components of the oxidative phosphorylation complex were reduced in SNU1041 and SNU1076 cell lines (Figure 5(e)). Thus, our data revealed that NTPAM treatment induces mitochondrial dysfunction in HNC cells through mitochondrial damage.

### 3.6. NTPAM-Induced Enhancement of mtROS Is Associated with Overexpression of ATF4 and CHOP via Mitochondrial Damage

**Figure 6:**
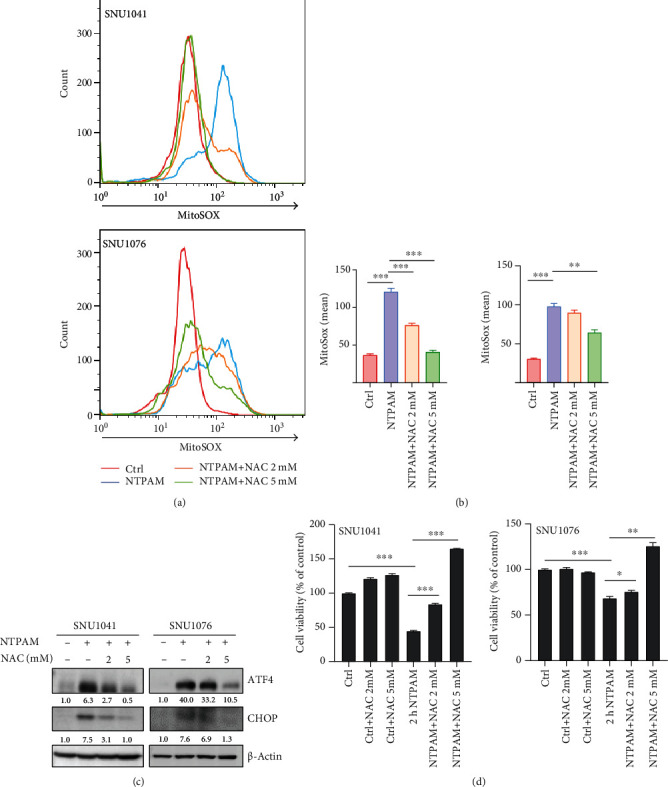
NTPAM-induced enhancement of mtROS is associated with overexpression of ATF4 and CHOP via mitochondrial damage. (a, b) mtROS expression levels after NTPAM and NAC treatments were evaluated by measurement of mitochondrial superoxide. mtROS levels increased significantly with NTPAM treatment in SNU1041 and SNU1076 cell lines. This enhanced mtROS expression was significantly reduced by application of the ROS scavenger NAC. (c) After treatment with NAC, changes in ATF4 and CHOP protein levels were evaluated by Western blotting. NTPAM treatment increased ATF4 and CHOP levels, but these changes were mitigated by NAC treatment. (d) WST-1 assays showed that the reduced cell viability after NTPAM treatment was alleviated by NAC treatment. ^∗∗^*p* ≤ 0.01; ^∗∗∗^*p* ≤ 0.001.

In a previous study, NTP induced the accumulation of mtROS and caused HNC cell death [15]. Therefore, we hypothesized that mtROS accumulation occurred following NTPAM treatment and may be connected to the overexpression of ATF4 and CHOP. To assess changes in mtROS, MitoSOX was used to measure mitochondrial peroxide levels, which significantly increased after NTPAM treatment (Figures 6(a) and 6(b)). NAC has antioxidant effects and scavenges mtROS, including intracellular ROS. We found that the NTPAM-induced enhancement of mtROS levels was mitigated by NAC treatment (Figures 6(a) and 6(b)). In addition, NTPAM-induced enhancements of ATF4 and CHOP protein levels were both reduced in an NAC concentration-dependent manner (Figure 6(c)). Finally, cell viability was significantly reduced after NTPAM treatment, but this effect was reversed by NAC treatment (Figure 6(d)).

### 3.7. NTPAM Suppressed HNC Progression in a Mouse Xenograft Model

**Figure 7:**
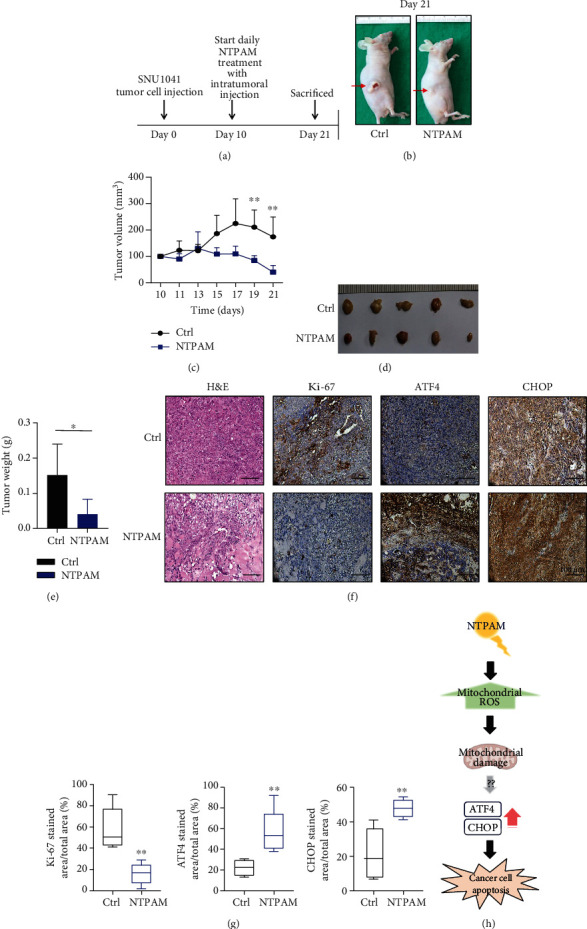
NTPAM suppressed HNC progression in a mouse xenograft model. (a) Schematic diagram of in vivo experiment. (b) Gross appearance of mouse xenograft model with tumor formation. (c) Volume measured during the experimental period. Compared with the control group, tumor volumes were reduced in the NTPAM-treated group. (d) Gross morphology of tumor excised from xenograft models after mice had been sacrificed. (e) Weight measurements of excised tumors. After NTPAM treatment, the tumor weight was significantly lower than in control tumors. (f, g) Histological and immunohistochemical analysis of tumor tissue after NTPAM treatment. In tumor tissues, low expression levels of Ki-67 and high expression levels of ATF4 and CHOP were observed in the NTPAM-treated group, compared with the normal control group. (h) Schematic diagram of the proposed mechanism of NTPAM anticancer effects on HNC cells. ^∗^*p* ≤ 0.05; ^∗∗^*p* ≤ 0.01.

To evaluate the in vivo effects of NTPAM on HNC, experiments were performed using a mouse xenograft model established by subcutaneous injection of SNU1041 cells (5 × 10^6^ cells/mL). After 10 days, tumor growth was visually confirmed, and NTPAM (100 *μ*L) was then injected intratumorally once daily (Figures 7(a) and 7(b)). Changes in tumor volume were recorded during the experiment, and mice were sacrificed on the 21st day (Figures 7(a) and 7(b)). Compared to the NTPAM-treated group, the tumor volume in the control group was significantly larger during the experiment period (Figure 7(c)). On the 21st day, analysis of tumors harvested from sacrificed mice showed that the tumor volume and weight were significantly lower in the NTPAM-treated group (Figures 7(c)–7(e)).

Hematoxylin and eosin (H&E) staining of tumor tissue showed excessive cell proliferation of tumor cells in the control group, whereas hyalinization was observed due to the death of cancer cells in the NTPAM-treated group (Figure 7(f)). Immunohistochemical staining was used to evaluate the expression levels of Ki-67, ATF4, and CHOP in tumor tissue. Ki-67 expression was high in the control group. In contrast, the expression levels of ATF4 and CHOP were elevated in tumor tissue after NTPAM treatment. These results indicated that NTPAM attenuated the progression of HNC in vivo through elevated expression levels of ATF4 and CHOP (Figures 7(f) and 7(g)).

The findings in this study suggested that NTPAM induces mtROS enhancement, resulting in mitochondrial damage and increased HNC cell apoptosis through the ATF4/CHOP signaling pathway (Figure 7(h)).

## 4. Discussion

Despite progress in the diagnosis and treatment of HNC, its incidence and mortality continue to increase [23, 24]. Surgery, chemotherapy, and radiation therapy for advanced HNC are applied as single or combination treatments, but there has been no substantial improvement in the 5-year survival rates of some HNC types [2]. Precision medicine has been used to develop targeted therapeutics in recent years. However, there are several limitations such as treatment resistance, side effects, and tumor heterogeneity [25–27]. A new type of anticancer treatment approach is needed to overcome these therapeutic limitations.

Plasma medicine is rapidly becoming an important potential treatment field. NTP is effective for regenerating damaged tissue epithelium and thus protects normal tissue in various disease models [7, 9, 10, 28]. This suggests that the use of NTP as an alternative therapy can overcome the limitations of existing anticancer treatment due to toxicity in normal cells.

In particular, NTPAM is useful because it has an anticancer effect similar to that of existing NTP and can be directly applied to tumors [29–31]. The anticancer effects of NTP are broadly related to mechanisms such as apoptosis induction through ROS generation and cytoskeletal modulation [6, 31, 32], but the detailed underlying mechanisms are unclear. To our knowledge, there have been few studies concerning the mechanism by which NTPAM mediates its anticancer effects. Therefore, our study is important in that it confirms the anticancer effects of NTPAM in HNC cell lines and provides transcriptomics-related insights concerning the underlying mechanisms of these anticancer effects.

ATF4 is a transcription factor expressed in the context of sustained ER stress, which induces adaptation to stress conditions [20, 33]. It is also related to the regulation of mitochondrial stress [22]. CHOP regulates the expression of proapoptosis-related genes and is activated by ATF4 [34, 35]. The excessive accumulation of mtROS induces mitochondrial permeability transition, thus causing damage through osmotic expansion of the mitochondrial matrix [36, 37]. This mechanism is an important component of mitochondrial targeted therapy [36]. Here, we found that NTPAM induced the enhancement of mtROS in HNC cells, as well as mitochondrial damage. IPA analysis revealed that the expression patterns of ATF4 and CHOP, major upstream regulators of the NTPAM-induced anticancer effect, were altered by treatment with NAC, a ROS scavenger. Therefore, we hypothesized that the anticancer mechanism of NTPAM involved enhanced mitochondrial stress and mitochondrial damage due to the accumulation of mtROS, which led to the overexpression of both ATF4 and CHOP, as well as apoptosis. We experimentally validated this hypothesis both in vitro and in vivo. However, additional studies are required to undercover the intermediate pathway through which mitochondrial damage causes overexpression of ATF4 and CHOP.

Mitochondria are organelles responsible for energy metabolism, cellular redox/calcium homeostasis, and cell death regulation in normal cells [38, 39]. Furthermore, mitochondria are also important mediators of tumorigenesis because they regulate apoptosis and are thus involved in the formation of neoplasms (through the alteration of mitochondrial function) [36, 40]. Cancer cells undergo metabolic reprogramming through changes in mitochondrial function, and tumorigenesis is promoted by changes in cancer cell-specific metabolites [41]. Mitochondrial targeted therapy is a unique approach that differs considerably from existing anticancer drugs and can enable individualized treatment of patients with carcinomas [36]. The anticancer effects of NTPAM are closely related to the regulation of ATF4 and CHOP activities, in which transcriptome analysis revealed that they were related to the mitochondrial stress response. Experimental analysis showed that these effects were mediated by mitochondrial damage. Therefore, NTPAM is presumably suitable as a cancer-specific mitochondrial targeting agent. To establish the generalizability of this mechanism, additional research is needed to determine whether the same phenomenon appears in other cancer types.

Here, we performed an in vivo experiment involving an intratumoral injection method, which showed positive results supporting its potential use in clinical practice. In particular, NTPAM can be applied to HNC tumors in various forms such as gargle or spray, in addition to a liquid injection method. Therefore, NTPAM offers an alternative treatment for HNC cancer that is refractory to existing treatment. However, for future clinical applications, large-scale experiments with medium and large animals must be performed. Further studies are also needed to optimize the dose of NTPAM and establish its safety characteristics.

## 5. Conclusions

In conclusion, NTPAM induces the accumulation of mtROS in HNC cells. Mitochondrial damage caused by this accumulation of mtROS induces the overexpression of both ATF4 and CHOP, thereby increasing apoptosis. NTPAM offers a new alternative therapy that can overcome limitations involving toxic effects in normal cells and resistance to conventional cancer therapy.

## Data Availability

The data used to support the findings of this study are available from the corresponding author upon request.

## Supplementary Materials

Supplementary 1 Figure S1: nonthermal plasma-activated media (NTPAM) generation system and specification. Figure S2: oxygen consumption rate (OCR) measurement in human fibroblasts after NTPAM treatment.

Supplementary 2 Supplementary Table S1: PCR primer sequence. Supplementary Table S2: siRNA sequence.

## References

[1] Bray F., Ferlay J., Soerjomataram I., Siegel R. L., Torre L. A., Jemal A. (2018). Global cancer statistics 2018: GLOBOCAN estimates of incidence and mortality worldwide for 36 cancers in 185 countries. *CA: a Cancer Journal for Clinicians*.

[2] Pulte D., Brenner H. (2010). Changes in survival in head and neck cancers in the late 20th and early 21st century: a period analysis. *The Oncologist*.

[3] (2018). By the Numbers: Cancer Mortality, 2011-2015. *Cancer Discovery*.

[4] Zhang W., Hu X., Shen Q., Xing D. (2019). Mitochondria-specific drug release and reactive oxygen species burst induced by polyprodrug nanoreactors can enhance chemotherapy. *Nature Communications*.

[5] Haertel B., von Woedtke T., Weltmann K. D., Lindequist U. (2014). Non-thermal atmospheric-pressure plasma possible application in wound healing. *Biomolecules & therapeutics*.

[6] Chang J. W., Kang S. U., Shin Y. S. (2014). Non-thermal atmospheric pressure plasma inhibits thyroid papillary cancer cell invasion via cytoskeletal modulation, altered MMP-2/-9/uPA activity. *PLoS One*.

[7] Lee M. H., Lee Y. S., Kim H. J., Han C. H., Kang S. U., Kim C. H. (2019). Non-thermal plasma inhibits mast cell activation and ameliorates allergic skin inflammatory diseases in NC/Nga mice. *Scientific Reports*.

[8] Ke Z., Huang Q. (2016). Haem-assisted dityrosine-cross-linking of fibrinogen under non-thermal plasma exposure: one important mechanism of facilitated blood coagulation. *Scientific Reports*.

[9] Won H. R., Kang S. U., Kim H. J., Jang J. Y., Shin Y. S., Kim C. H. (2018). Non-thermal plasma treated solution with potential as a novel therapeutic agent for nasal mucosa regeneration. *Scientific Reports*.

[10] Won H. R., Song E. H., Won J. E. (2019). Liquid-type non-thermal atmospheric plasma ameliorates vocal fold scarring by modulating vocal fold fibroblast. *Experimental Biology and Medicine (Maywood)*.

[11] Vandamme M., Robert E., Lerondel S. (2012). ROS implication in a new antitumor strategy based on non-thermal plasma. *International journal of cancer*.

[12] Hou J., Ma J., Yu K. N. (2015). Non-thermal plasma treatment altered gene expression profiling in non-small-cell lung cancer A549 cells. *BMC Genomics*.

[13] Liedtke K. R., Bekeschus S., Kaeding A. (2017). Non-thermal plasma-treated solution demonstrates antitumor activity against pancreatic cancer cells *in vitro* and *in vivo*. *Scientific Reports*.

[14] Choi J. S., Kim J., Hong Y. J. (2017). Evaluation of non-thermal plasma-induced anticancer effects on human colon cancer cells. *Biomedical optics express*.

[15] Kang S. U., Cho J. H., Chang J. W. (2014). Nonthermal plasma induces head and neck cancer cell death: the potential involvement of mitogen-activated protein kinase-dependent mitochondrial reactive oxygen species. *Cell death & disease*.

[16] Saadati F., Mahdikia H., Abbaszadeh H. A., Abdollahifar M. A., Khoramgah M. S., Shokri B. (2018). Comparison of direct and indirect cold atmospheric-pressure plasma methods in the B_16_F_10_ melanoma cancer cells treatment. *Scientific Reports*.

[17] Tanaka H., Nakamura K., Mizuno M. (2016). Non-thermal atmospheric pressure plasma activates lactate in Ringer's solution for anti-tumor effects. *Scientific Reports*.

[18] Tanaka H., Mizuno M., Ishikawa K. (2012). Cell survival and proliferation signaling pathways are downregulated by plasma-activated medium in glioblastoma brain tumor cells. *Plasma Medicine*.

[19] Jung S. N., Oh C., Chang J. W. (2021). EGR1/GADD45*α* activation by ROS of non-thermal plasma mediates cell death in thyroid carcinoma. *Cancers (Basel)*.

[20] Rozpedek W., Pytel D., Mucha B., Leszczynska H., Diehl J. A., Majsterek I. (2016). The role of the PERK/eIF2*α*/ATF4/CHOP signaling pathway in tumor progression during endoplasmic reticulum stress. *Current molecular medicine*.

[21] Hunt R. J., Granat L., McElroy G. S., Ranganathan R., Chandel N. S., Bateman J. M. (2019). Mitochondrial stress causes neuronal dysfunction via an ATF4-dependent increase in L-2-hydroxyglutarate. *The Journal of Cell Biology*.

[22] Quirós P. M., Prado M. A., Zamboni N. (2017). Multi-omics analysis identifies ATF4 as a key regulator of the mitochondrial stress response in mammals. *Journal of Cell Biology*.

[23] Simard E. P., Torre L. A., Jemal A. (2014). International trends in head and neck cancer incidence rates: differences by country, sex and anatomic site. *Oral oncology*.

[24] Global Burden of Disease Cancer Collaboration, Fitzmaurice C., Abate D. (2019). Global, regional, and national cancer incidence, mortality, years of life lost, years lived with disability, and disability-adjusted life-years for 29 cancer groups, 1990 to 2017: a systematic analysis for the Global Burden of Disease study. *JAMA oncology*.

[25] Lee Y. T., Tan Y. J., Oon C. E. (2018). Molecular targeted therapy: treating cancer with specificity. *European journal of pharmacology*.

[26] Widakowich C., de Castro G., de Azambuja E., Dinh P., Awada A. (2007). Review: side effects of approved molecular targeted therapies in solid cancers. *The Oncologist*.

[27] Loges S., Schmidt T., Carmeliet P. (2010). Mechanisms of resistance to anti-angiogenic therapy and development of third-generation anti-angiogenic drug candidates. *Genes Cancer*.

[28] Lee Y. S., Lee M. H., Kim H. J., Won H. R., Kim C. H. (2017). Non-thermal atmospheric plasma ameliorates imiquimod-induced psoriasis-like skin inflammation in mice through inhibition of immune responses and up- regulation of PD-L1 expression. *Scientific reports*.

[29] Chauvin J., Judée F., Yousfi M., Vicendo P., Merbahi N. (2017). Analysis of reactive oxygen and nitrogen species generated in three liquid media by low temperature helium plasma jet. *Scientific reports*.

[30] Yan D., Nourmohammadi N., Bian K., Murad F., Sherman J. H., Keidar M. (2016). Stabilizing the cold plasma-stimulated medium by regulating medium's composition. *Scientific reports*.

[31] Anzai K., Aoki T., Koshimizu S., Takaya R., Tsuchida K., Takajo T. (2019). Formation of reactive oxygen species by irradiation of cold atmospheric pressure plasma jet to water depends on the irradiation distance. *Journal of clinical biochemistry and nutrition*.

[32] Iuchi K., Morisada Y., Yoshino Y. (2018). Cold atmospheric-pressure nitrogen plasma induces the production of reactive nitrogen species and cell death by increasing intracellular calcium in HEK293T cells. *Archives of biochemistry and biophysics*.

[33] Schonthal A. H. (2012). Endoplasmic reticulum stress: its role in disease and novel prospects for therapy. *Scientifica (Cairo)*.

[34] Dey S., Baird T. D., Zhou D., Palam L. R., Spandau D. F., Wek R. C. (2010). Both Transcriptional Regulation and Translational Control of ATF4 Are Central to the Integrated Stress Response. *Journal of Biological Chemistry*.

[35] Oyadomari S., Mori M. (2004). Roles of CHOP/GADD153 in endoplasmic reticulum stress. *Cell Death and Differentiation*.

[36] Fulda S., Galluzzi L., Kroemer G. (2010). Targeting mitochondria for cancer therapy. *Nature Reviews. Drug Discovery*.

[37] Kroemer G., Galluzzi L., Brenner C. (2007). Mitochondrial membrane permeabilization in cell death. *Physiological Reviews*.

[38] Wang S. F., Chen S., Tseng L. M., Lee H. C. (2020). Role of the mitochondrial stress response in human cancer progression. *Experimental Biology and Medicine*.

[39] Galluzzi L., Joza N., Tasdemir E. (2008). No death without life: vital functions of apoptotic effectors. *Cell Death and Differentiation*.

[40] Gogvadze V., Orrenius S., Zhivotovsky B. (2008). Mitochondria in cancer cells: what is so special about them?. *Trends in cell biology*.

[41] Vyas S., Zaganjor E., Haigis M. C. (2016). Mitochondria and cancer. *Cell*.

