# Supplementary material for: Head and Neck Cancer Cell Death due to Mitochondrial Damage Induced by Reactive Oxygen Species from Nonthermal Plasma-Activated Media: Based on Transcriptomic Analysis
Source: Oxid Med Cell Longev. 2021 Jul 6;2021:9951712. doi: 10.1155/2021/9951712 (PMC8281449; doi:10.1155/2021/9951712)
Supplement: Supplementary 1 — Figure S1: nonthermal plasma-activated media (NTPAM) generation system and specification. Figure S2: oxygen consumption rate (OCR) measurement in human fibroblasts after NTPAM treatment. [file 9951712.f1.pdf]

Supplement Figure 1. Non-thermal plasma activated media (NTPAM) generation system and specification.

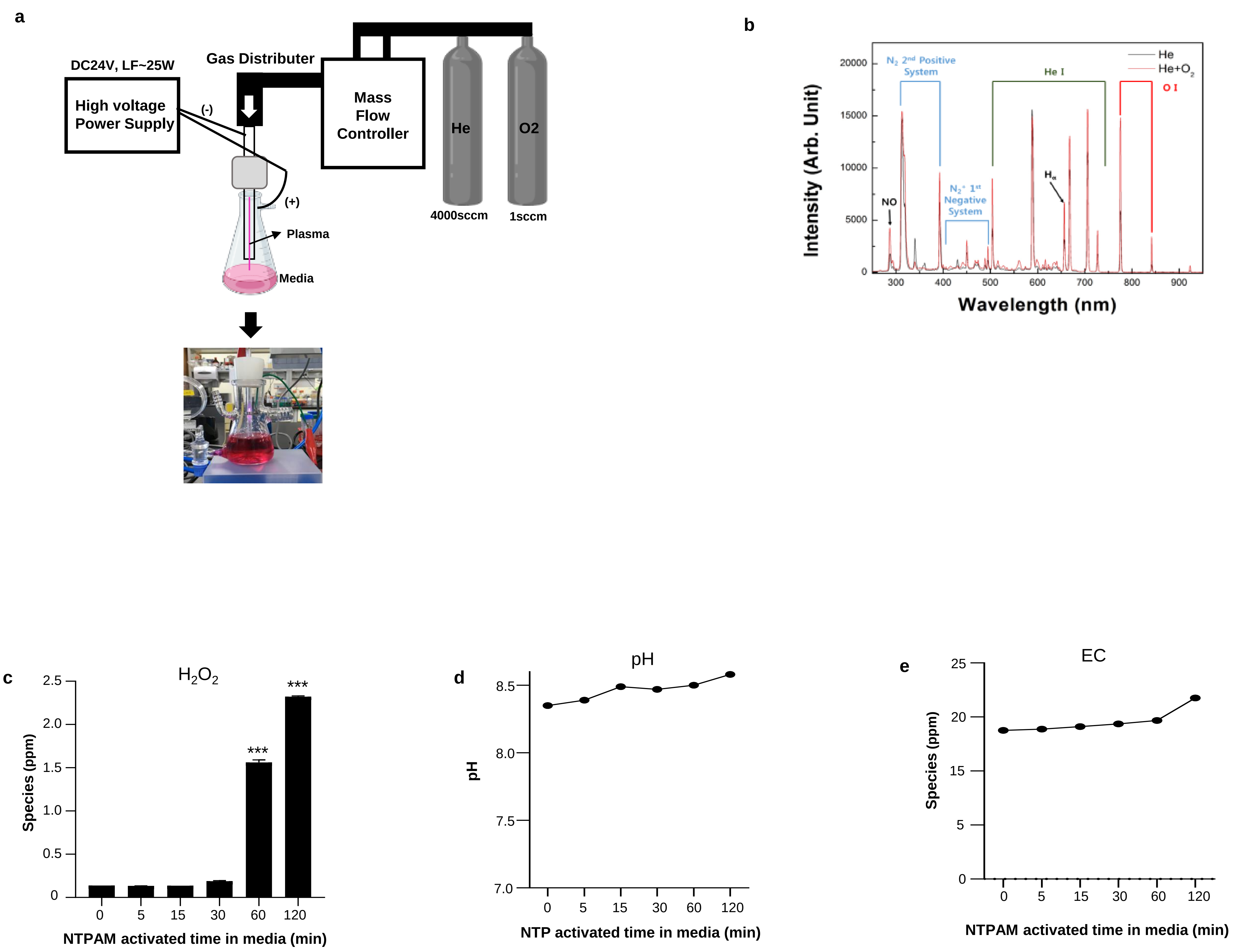

Figure S1. Non-thermal plasma activated media (NTPAM) generation system and specification. (a) Schematic illustration and photo of the NTPAM generation system. DC=Direct Current; LF=Low Frequency; SCCM=Standard Cubic Centimeter per Minute. (b) Optical emission spectra of the NTP according to the electric intensity (2 or 4 kV) in the range of 280–920 nm. (c) Change of H_2O_2 value with NTP activated time in NTPAM. (d) Change of pH value with NTP activated time in NTPAM. (e) NTP activated time-dependent degree of electric conductivity (EC) in NTPAM.

Supplement Figure 2. Oxygen consumption rate (OCR) measurement in human fibroblasts after NTPAM treatment.

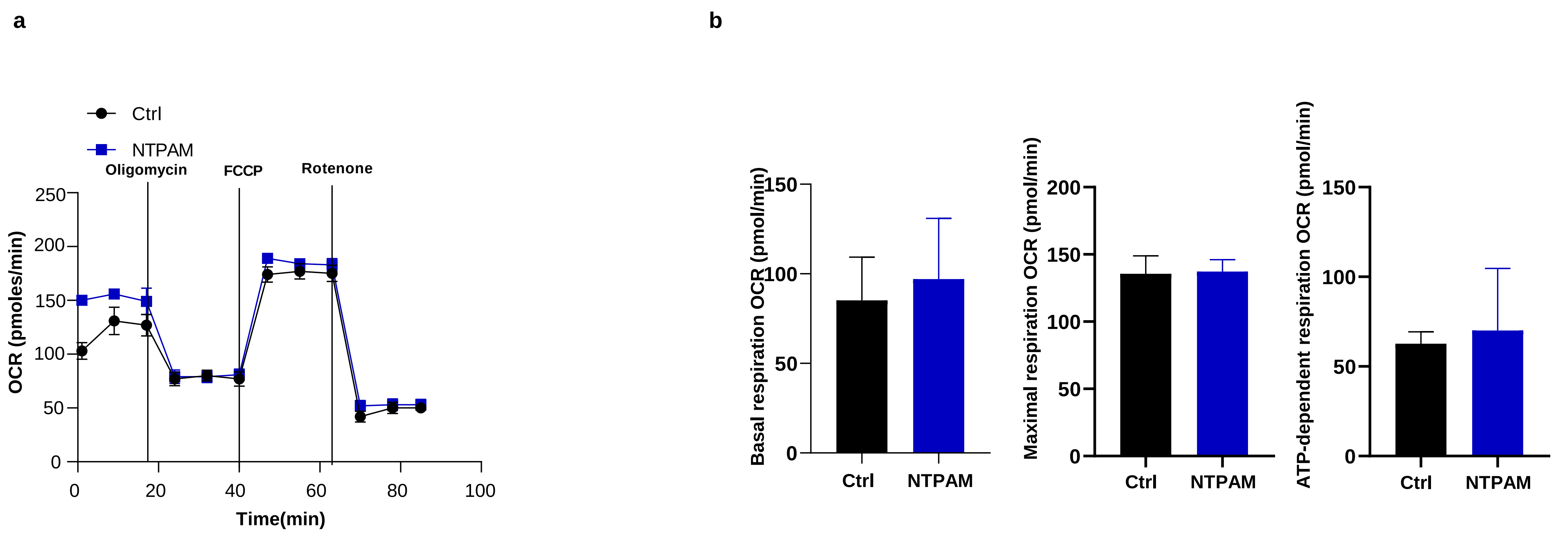

Figure S2. Oxygen consumption rate (OCR) measurement in human fibroblasts after NTPAM treatment. (a)(b) There was no significant change in OCR after NTPAM treatment in human fibroblast.
