# Supplementary material for: Head and Neck Cancer Cell Death due to Mitochondrial Damage Induced by Reactive Oxygen Species from Nonthermal Plasma-Activated Media: Based on Transcriptomic Analysis
Source: Oxid Med Cell Longev. 2021 Jul 6;2021:9951712. doi: 10.1155/2021/9951712 (PMC8281449; doi:10.1155/2021/9951712)
Supplement: Supplementary 2 — Supplementary Table S1: PCR primer sequence. Supplementary Table S2: siRNA sequence. [file 9951712.f2.docx]

**Supplementary Materials**

**Supplementary Table. S1: PCR primer sequence**

| Gene | Primer | Sequence (5’-3’) | Size(bp) |
| --- | --- | --- | --- |
| Human ATF4 | Forward | 5’-CTG GAG AGA AGA TGG TAG CAG CAA-3’ | 112 |
|  | Reverse | 5’-GCC CTC TTC TTC TGG CGG TA-3’ |  |
| Human CHOP | Forward | 5’-TGG AAG CCT GGT ATG AGG AC-3’ | 170 |
|  | Reverse | 5’-TGT GAC CTC TGC TGG TTC TG-3’ |  |
| Human GAPCH | Forward | 5’-GTC TCC TCT GAC TTC AAC AGC G-3’ | 131 |
|  | Reverse | 5’-ACC ACC CTG TTG CTG TAG CCA A-3’ |  |

**Supplementary Table. S2: siRNA sequence**

| Gene | siRNA pool | Sequence |
| --- | --- | --- |
| ATF4 | sc-35112A | 5’-CCACUCCAGAUCAUUCCUUtt-3’ |
|  |  | 5’-AAGGAAUGAUCUGGAGUGGtt-3’ |
|  | sc-35112B | 5’-GGAUAUCACUGAAGGAGAUtt-3’ |
|  |  | 5’-AUCUCCUUCAGUGAUAUCCtt-3’ |
|  | sc-35112C | 5’-GUGAGAAACUGGAUAAGAAtt-3’ |
|  |  | 5’-UUCUUAUCCAGUUUCUCACtt-3’ |
